# Design and Performance Evaluation of a “Fixed-Point” Spar Buoy Equipped with a Piezoelectric Energy Harvesting Unit for Floating Near-Shore Applications †

**DOI:** 10.3390/s21051912

**Published:** 2021-03-09

**Authors:** Damiano Alizzio, Marco Bonfanti, Nicola Donato, Carla Faraci, Giovanni Maria Grasso, Fabio Lo Savio, Roberto Montanini, Antonino Quattrocchi

**Affiliations:** 1 Department of Engineering, University of Messina, C.da Di Dio, 98166 Messina, Italy; nicola.donato@unime.it (N.D.); carla.faraci@unime.it (C.F.); roberto.montanini@unime.it (R.M.); 2 Department of Electrical, Electronic and Computer Engineering, University of Catania, Via S. Sofia, 54, 95100 Catania, Italy; marco.bonfanti@unict.it (M.B.); ggrasso@dii.unict.it (G.M.G.); 3 Department of Civil Engineering and Architecture, University of Catania, Via S. Sofia, 54, 95100 Catania, Italy; flosavio@diim.unict.it

**Keywords:** spar-buoy, energy harvesting, fixed-point, near-shore buoy, piezo patch transducers

## Abstract

In the present work, a spar-buoy scaled model was designed and built through a “Lab-on-Sea” unit, equipped with an energy harvesting system. Such a system is based on deformable bands, which are loyal to the unit, to convert wave motion energy into electricity by means of piezo patch transducers. In a preliminary stage, the scaled model, suitable for tests in a controlled ripples-type wave motion channel, was tested in order to verify the “fixed-point” assumption in pitch and roll motions and, consequently, to optimize energy harvesting. A special type of structure was designed, numerically simulated, and experimentally verified. The proposed solution represents an advantageous compromise between the lightness of the used materials and the amount of recoverable energy. The energy, which was obtained from the piezo patch transducers during the simulations in the laboratory, was found to be enough to self-sustain the feasible on-board sensors and the remote data transmission system.

## 1. Introduction

Nowadays, the monitoring of marine habitat is a topic of great relevance [1]. These environments are particularly susceptible to human activity, in terms of industrial and civil development. Therefore, acquiring information on sufficiently large spatial and temporal enough scales is essential for guaranteeing effective monitoring and, for such reason, to produce solutions aimed at reducing anthropogenic impact on ecosystems. These include monitoring the concentration of pollutants in both natural and artificial reservoirs and near sewage, as well as the detection of microplastics and their concentration in lagoon or open sea waters [2,3]. In addition, there are also multiple civil protection purposes, including the possibility of detecting anomalous waves or tsunamis that can occur on the coasts [4]. The scientific literature is rich of solutions that involve the use of floating devices, equipped with a wide range of sensors, for environmental monitoring and adequate telecommunication interfaces. The most sophisticated devices employ energy recovery systems for self-maintenance (e.g., energy harvesting), so that they can be used offshore [5]. Observation platforms include various types: on site, equipped with remote instrumentation, operating on the surface (on board an oceanographic ship [6], surface buoys [7,8], floating buoys [9,10], buoys towed by boats [11]), active on the seabed (autonomous underwater vehicles AUV [12], remote control vehicles ROV [13], networks of underwater buoys [14]), and with satellite monitoring [15]. Usually, piezoelectric patch transducers (PPTs) are employed as both actuators [16], to convert electrical voltage into strain, and sensors, in order to measure displacements and strains [17]. Recently, PPTs have been largely reconsidered to develop vibration energy harvesting devices, thanks to their limited size and weight [18]. The choice of the type of buoy and equipment to be adopted is mainly determined by the characteristics of the environment to be investigated (depth of the waters, peculiarities of the waves, etc.) and the required space-time coverage [19].

In this context, the subject of our paper concerns some preparatory operations, which were previously exposed on the IMEKO MetroSea 2020 [20], for the future design of a multi-purpose “Lab on Sea” unit, able to perform energy storage automatically from waves and equipped with a communication network for connecting to any IT infrastructure. The idea of building a unit, supplied with power devices from renewable sources to minimize the implementation and maintenance costs of the entire data collection system and to ensure energy autonomy, was deemed to be convenient. The ultimate purpose of this investigation consists in designing additional elements of the unit that are able to perform energy harvesting. Thus, the target will be to develop a system with a set of deformable and wave-sensitive bands to convert the mechanical strain energy into electricity through piezoelectric patch transducers (PPT).

## 2. Preliminary Study

**Figure 1:**
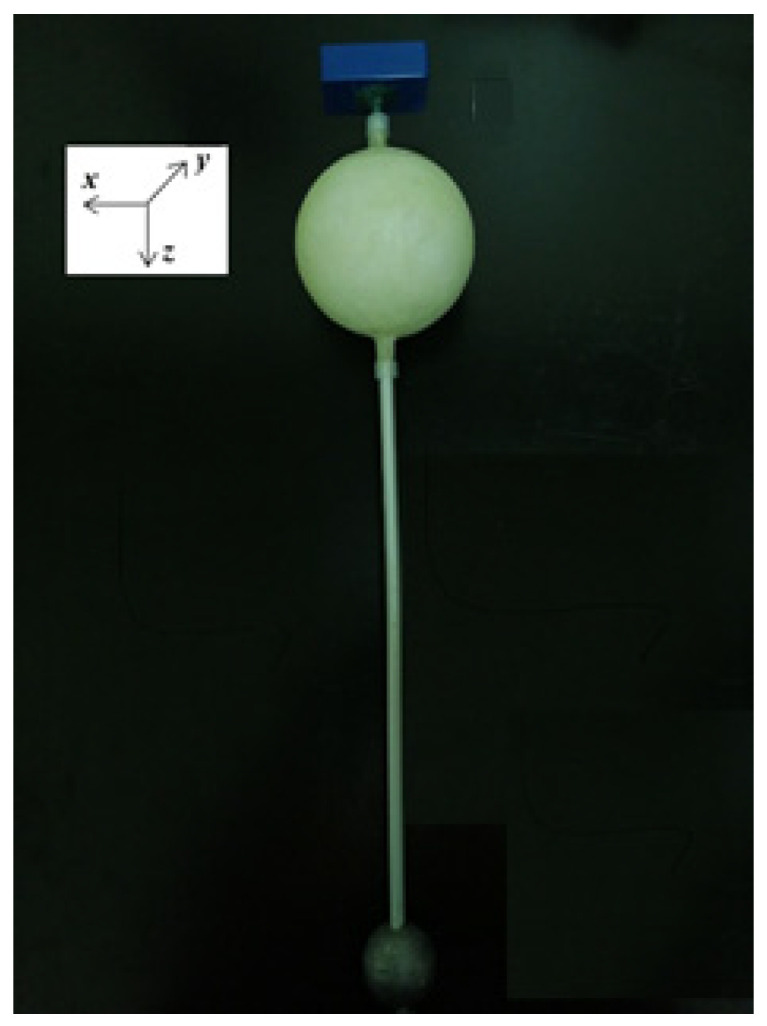
Spar buoy scaled model at “fixed-point” test configuration.

The spar buoy scaled model designed in the present paper consists of a spherical buoy with a central through hole, where a rigid rod is inserted. The rod has the dual purpose of supporting a stabilizing counterweight (ballast) in its immersed part and accommodating the sensors and electronics on the top (Figure 1).

## 2.1. “Fixed-Point” Spar Buoy

**Figure 2:**
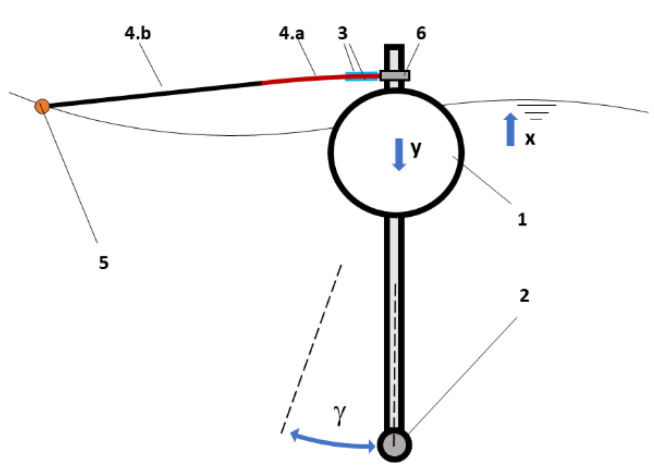
Scheme of the energy conversion apparatus for the Lab on sea unit: 1. spar buoy; 2. ballast; 3. piezoelectric patch transducers (PPTs); 4. band (a) deformable part, (b) rigid part; 5. external float; and, 6. rod grip. The vertical direction of the wave motion is indicated with *x*, while *y* is the vertical displacement of the spay buoy and *γ* is its angular oscillation.

Because piezoelectric elements are better performing at high frequencies, these abovementioned bands will be sensitive to ripples-type waves. It will be necessary to maximize the strain by constraining the unit to remain fixed with respect to the wave motion in order to amplify the input of the transducers. Hereafter, such a condition is named “fixed-point” (Figure 2).

Subsequently, the PPTs will be joined on the surface of these bands close to the central body, in which the strain that is caused by the wave motion is expected to be maximum along the band axis. The presence of the buoy central body would affect the motion of the terminal float if it overlaps between the wave front and the float. The float must be placed at not less than three lengths overall (LOA) in order to minimize this interference [21,22]. Because the scaled buoy diameter was 100 mm, that minimum distance should have been 300 mm, at least. Therefore, the length of buoy-float connection arm was chosen equal to 350 mm. If the system respects the “fixed-point” conditions that were ensured by the fluid dynamic response of the central body of the buoy, the strain of the bands is achieved, whereas the opposite end is free to move with the wave motion. Consequently, a float with a low mass/buoyancy ratio will be used on the free end of the bands in order to capture high frequency wave motions. Finally, an evaluation of the amount of the energy that can be extracted for a typical sea wave motion will be made.

In the preliminary study of this paper, the dynamic behavior of a spar buoy scaled model that had undergone a known wave motion was investigated in order to verify that the “fixed-point” condition was satisfied. The scaled model can be assimilated to a 2 DOFs mass-spring-damper system and, therefore, it was sized to make both vertical and angular oscillations negligible [23]. As known, a mass-spring-damper system of mass (*m*), stiffness (*k*), and viscosity (*β*), which was placed on a vibrating body (such as a fluid subject to wave motion) under an oscillation *x = X*_0_ *sin*(*ωt*) with respect to a fixed reference system, is characterized by the Equation (1) [24]:

*mÿ* + *βẏ* + *ky* = *mω*^2^*sin*(*ωt*) (1)

The ratio (Equation (2)) between the magnitudes of the dynamical response (*Y*_0_) and forcing excitation (*X*_0_) depends on the pulsation of the excitation (*ω*), the pulsation of the system (*ω*_0_ *=* (*k/m*)^1/2^), the viscosity, and the critical value of viscosity (*β_cr_* = 2(*k m*)^1/2^):

*Y*_0_*/X*_0_ = *λ*^2^/((1 *− λ*^2^)^2^ + *λ*^2^*τ*^2^)^1/2^ (2)

and the phase angle (*φ*) (Equation (3)) between the dynamical response and forced excitation is given by:

*φ* = *tan*^−1^(−2*τλ*/(1 − *λ*^2^)) (3)

where *λ = ω/ω*_0_ and *τ = β/β_cr_*. The aim is creating a system with high values of *λ*, so that the ratio between the amplitudes tends to 1 and the phase angle to *π*, regardless of *τ*. This leads to a response having an amplitude that is equal to that of the excitation but in the counter-phase (i.e., the buoy is integral with the wave motion, but in the opposite direction).

The two DOFs represent the vertical displacement (*y*) and the angular oscillation (*γ*), as can be seen in Figure 1. However, the motion, as explained by Equations (1)–(3), is relative to a 1 DOF system, since the two oscillations (vertical and angular) can be considered to be independent from each other. As such, for the principle of superposition of effects, the above-mentioned dynamic is applicable to both types of oscillation.

**Figure 3:**
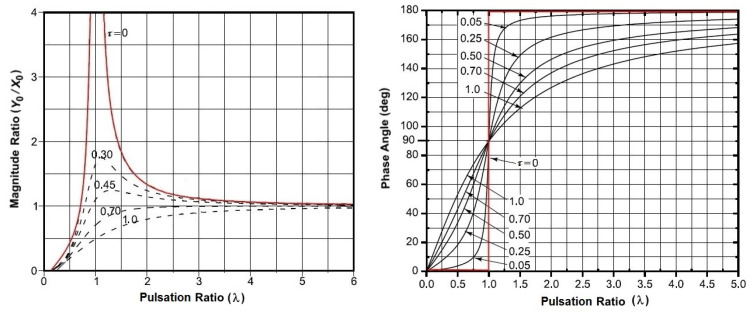
Magnitude ratio and phase of the response of a typical mass-spring-damper as a function of λ.

Figure 3 shows the effect of λ on the magnitude ratio and the phase of the response of a typical mass-spring-damper system.

Because, for λ > 5, the magnitude ratio and phase angle are independent of the viscosity of the system. Hence, the effect of damping can be neglected and the study of angular oscillations can be traced back to that of the harmonic motion of a simple pendulum of length (L) and natural frequency ωn = (g/L) 1/2.

## 2.2. Wave Motion Measurement

**Figure 4:**
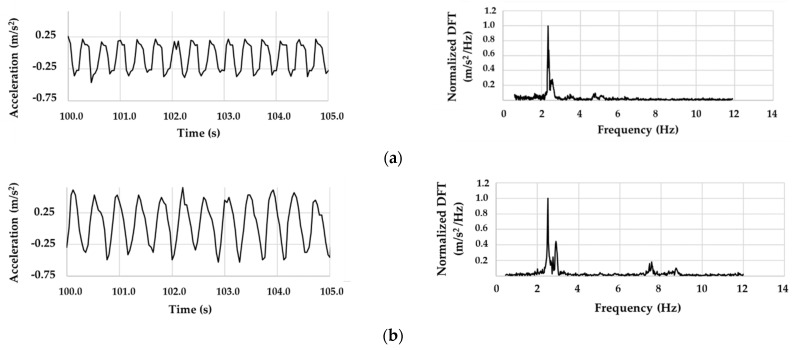
Comparison between acceleration and normalized DFT (**a**) at sea and (**b**) in artificial channel.

First of all, a preliminary experimental study was carried out both at sea and in an artificial channel for measuring the fundamental characteristics of the wave motion (amplitude and frequency). Indeed, the knowledge of wave frequencies is fundamental to correctly designing the spar buoy, above all in compliance with the required “fixed point” condition. It was found that the trend of the measured acceleration appears to be sufficiently repeatable in both tests. Furthermore, the frequency distributions are comparable, despite the different ways used to generate wave motion. The most representative frequency values acquired for ripples-type waves ranged from 1 to 3 Hz. Figure 4 shows the comparison between the vertical accelerations that were measured on sea near-shore (a) and under controlled conditions (artificial channel) (b), and their respective signals in the frequency domain obtained by means of a discrete Fourier transform (DFT).

## 2.3. Spar-Buoy Scaled Model

**Figure 5:**
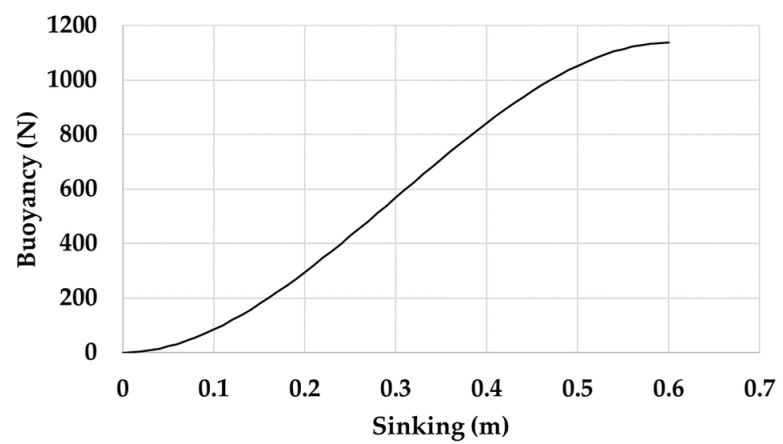
Buoyancy-sinking curve for the spar buoy.

From the analysis of measurements that were performed on sea and in the artificial channel, the required geometric dimensions that allows the unit to satisfy the “fixed-point” condition were analytically obtained. Table 1 reports the geometric and mechanical data for the spar buoy and its counterweight. Having imposed a value of 600 mm for the diameter (*d*) of the buoy, the choice of values that are useful for sinking (*S*) was made so as to fall within the linear section of the relative “buoyancy-sinking” curve. The latter is the correlation between the weight and the hydrostatic thrust for the spar buoy (Figure 5). In particular, *S* = 500 mm was adopted, which corresponds to a buoyancy (*B*) of nearly 1050 N, and the value of 100 mm was set for the emerged height of the spherical cap (*H_e_*). The ballast, which was also spherical in shape and made of lead (density of 11.34 kg/dm^3^), provided a counterweight of mass (*M*) equal to 57.5 kg and it was placed from the buoy center at a barycentric distance *L* = 2000 mm. When considering 2 Hz as the worst acceptable value for the oscillation frequency, the sizing of the buoy model provided sufficiently high values of *λ* (see Section 2.1) both as regards vertical oscillations (*λ_vert_* = 10.62) than the angular ones (*λ_ang_* = 5.67). These values of *λ* satisfy the “fixed-point” assumption and allow the simple pendulum approximation for the spar buoy, as shown in Figure 3.

However, a scaled model, suitable for tests in an artificial channel, was realized in order to verify experimentally that the “Lab on sea” unit will be able to satisfy the required “fixed-point” condition. Indeed, the water measurement system consisted of a channel to produce an established wave motion, having a wet section of 600 mm × 600 mm and length of 10 m. It was equipped with an air system for the generation of ripples-type waves and an optical measuring device for real-time acquisition of the wave height and frequency. Similar to the computation of Table 1, Table 2 shows the geometric and mechanical scaled data for the buoy model and its counterweight, achieved applying a linear scaled factor of 1/6. These reported values of *λ* satisfy the “fixed-point” assumption and allow the simple pendulum approximation for the spar buoy, as shown in Figure 3.

The model of the buoy built consists of a polystyrene sphere that was coated by deposition of polyester thermosetting epoxy fiber. The lead counterweight was made through a casting process on a dedicated plaster mold. The rigid rod for connecting the counterweight to the buoy was a cylindrical aluminum tubular with external diameter of 10 mm and thickness of 1 mm. On the top of the rod, a cylindrical seat accommodating a displacement sensor (iNEMO inertial module included in ST SensorTile.box, STMicroelectronics, Geneva, Switzerland) was realized. This sensor consisted of a triaxial gyroscope and a triaxial accelerometer. It was internally power supplied, and it allowed an easy reading of the measured quantities via Bluetooth communication and data logging on an internal SD card. The measurement range was selected between −500 and +500°/s for gyroscope and −39.24 and +39.24 m/s^2^ for accelerometer. Data sampling was performed at 104.0 Hz for both accelerometer and gyroscope signals, corresponding to a bandwidth cut-off frequency of 33.0 Hz according to sensors signal chain characteristics in the low energy mode. In this configuration, a gyroscope has a RMS rate noise value of 0.075°/s, while an accelerometer has a RMS rate noise value of 0.01962 m/s^2^. The sensitivity tolerance was ±1% on the measurement value both for the accelerometer and gyroscope. The temperature affects the accelerometer measurement in order of ±0.01% and the gyroscope measurement in the order of ±0.007%.

**Figure 6:**
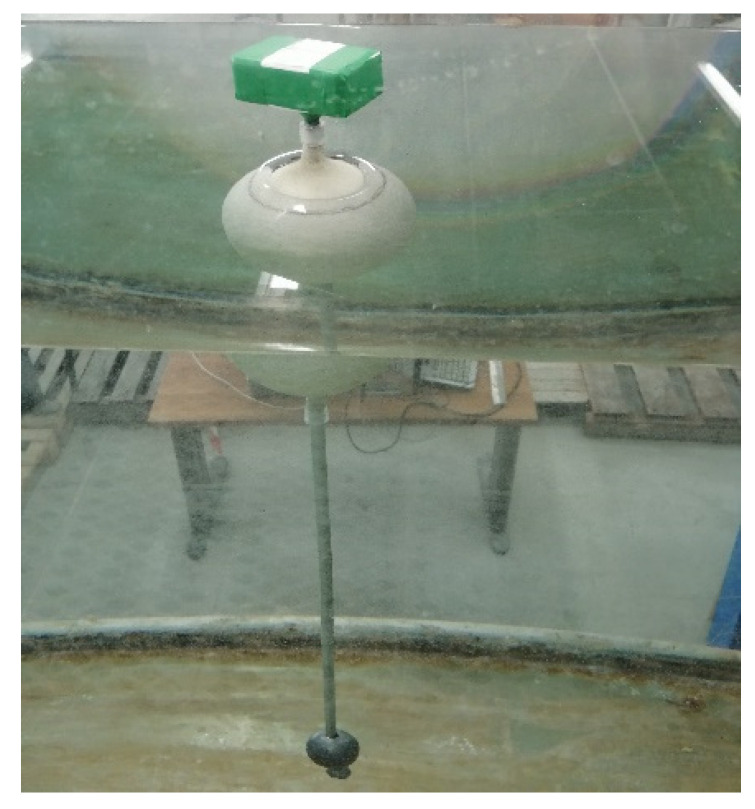
Spar buoy scaled model during test.

**Figure 7:**
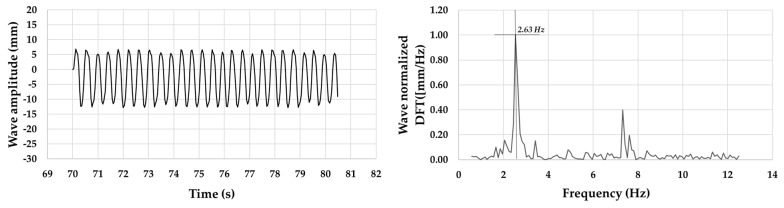
Wave amplitude and normalized DFT in artificial channel.

A dynamic buoyancy test of the scaled model in the above-mentioned controlled water channel was performed at a room temperature of 25 °C (Figure 6) [25]. There are various methodologies for evaluating the fluid dynamic behaviors in different applications [26]. The ripples-type waves were monitored via image acquisition and post-processed. For the acquisition, a camera Basler acA1300-30 gm GigE (Basler AG GmbH, Ahrensburg, Germany) with a CCD sensor (Sony ICX445, resolution of 1296 pixel × 966 pixel) and a 35 mm lens (C23-3520-2 M F2.0) were used. The motion reconstruction of the ripples-type waves was computed through NI Image Acquisition module on the LabView environment (National Instruments, Depew, NY, USA). The average value found for the imposed oscillation frequency was of 2.63 Hz, in line with the most representative frequency values of ripples-type waves, as shown in Figure 7. Acquisitions from both the on-board accelerometer and gyroscope were performed at 104.0 Hz. The ST SensorTile.box, which was mounted on the scaled buoy, returned values confirming the accomplishment of the required “fixed-point” condition.

## 3. Materials and Methods

### 3.1. Piezoelectric Energy Harvester

Specific experimental tests were carried out to investigate the amount of energy that can be accumulated over time with this type of design. For this purpose, a deformable band with two PPTs glued onto two opposite sides was manufactured and tested in lab. This condition is inevitable in ensuring that the device response is devoid of even the minimal fluctuations of the buoy, allowing a correct PPT calibration.

**Figure 8:**
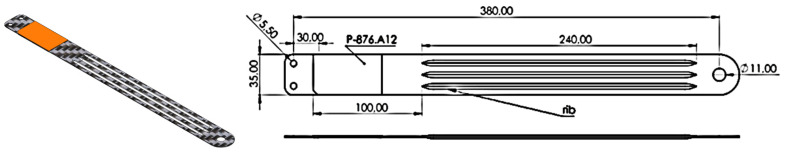
Geometrical characteristics of the deformable band.

A rectangular band of 1 mm in thickness and length equal to 380 mm was designed to satisfy the law in Section 1 [21,22]. Its effective length was 350 mm. At the free end, a 11 mm hole allowed for the insertion of the light float, able to follow the wave motion without altering it. Carbon fiber was the material that was chosen for the deformable band. Six epoxy resin ribs (240 × 5.0 × 1.2 mm^3^) were moulded along the two faces of the rectangular plate to limit the strain where the patches are not installed. Figure 8 shows the deformable band with two PPTs (mod. DuraAct P-876 A.12, Physik Instrumente GmbH, Karlsruhe, Germany) bonded one on each side. The piezo-active element of the PPT is encapsulated into a polymeric case, made of Kapton, with a thickness of 0.5 mm and then glued on the deformable band by means of a cyanoacrylate layer. This configuration guarantees that, at the investigated frequencies and displacements, a rigid constraint between PPT and deformable band is ensured, as reported in [18].

### 3.2. Numerical Simulation of Piezoelectric Energy Harvester

A time-dependent numerical simulation has been carried out using Solidworks to estimate the mechanical relevance of the above-mentioned system. Table 3 describes the physical and geometrical properties of the materials. Moreover, Table 3 displays Voronoy-Delaunay mesh characteristics, starting from the geometry reported in Figure 8. The boundary conditions applied to the mesh consisted of a fixed joint, imposed to the end of the band at the electrical contacts area of the PPT (30 mm from its position, as in Figure 8), and a sinusoidal vertical displacement at the free end. The characteristics of the sinusoidal displacement were of a magnitude equal to 10 mm and frequency equal to 2.63 Hz.

**Figure 9:**
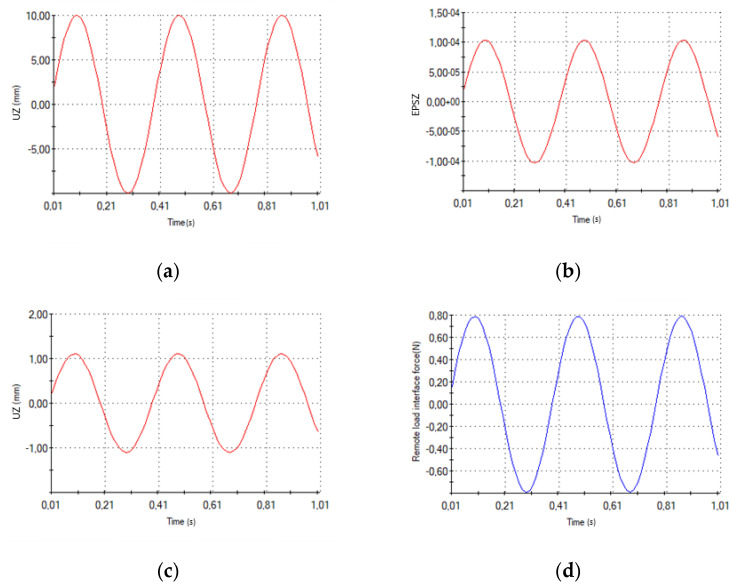
FEA screens from Solidworks: (**a**) imposed displacements at the free end over time, (**b**) Z-normal nodal averaged strain on upper PPT surface over time, (**c**) displacements along Z-axis at 100 mm from fixed joint cross-section over time, and (**d**) nodal averaged reaction force along Z-axis at 100 mm from fixed joint cross-section.

**Figure 10:**
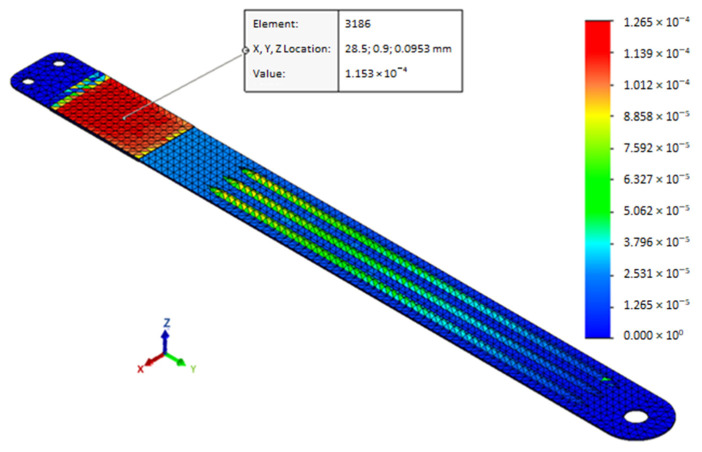
Z-axis normal strain corresponding to maximal imposed displacement. The measurement unit is mm/mm.

Figure 9 presents some of the characteristics that were computed through a finite element analysis (FEA). First of all, Figure 9a shows the displacement that was imposed to the free end. In Figure 9b and c, the mean strain into the plane normal to Z-axis on PPTs surfaces and the displacement detected in a nodal point at 100 mm from the fixed joint are shown, respectively. Figure 9d displays the reaction force trend that was evaluated on the cross-section of the deformable band at 100 mm from the fixed joint. FEA results exhibits a maximum strain along the deformable band on PPTs surfaces that equals 1.534 × 10^−4^ mm/mm (Figure 10), corresponding to the maximum displacement of the free end (10 mm). Strain magnitude values were estimated in this configuration to be sufficient to generate a suitable electrical response of PPTs, according to the scientific literature [20].

### 3.3. Experimental Setup

**Figure 11:**
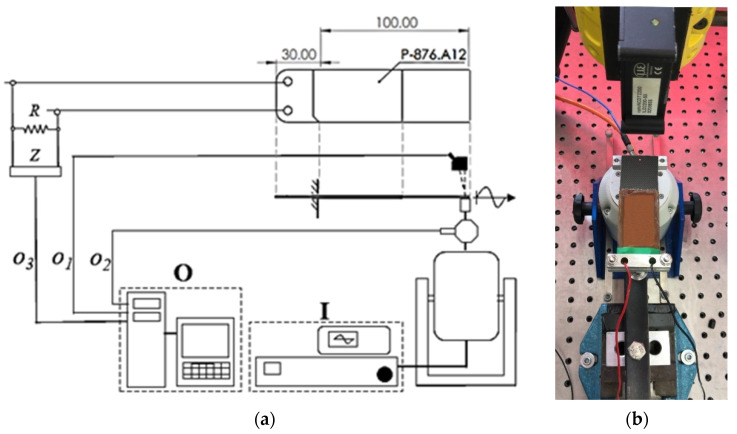
(**a**) Setup scheme (signal generator and amplifier (*I*), data acquisition system (*O*), linear displacement signal (*o*_1_), load cell signal (*o*_2_), PPT signal (*o*_3_), probe (*Z*), resistor (*R*)), and (**b**) experimental setup.

A rectangular band in carbon fiber with thickness of 1 mm, width of 35 mm, and length of 100 mm was built up in order to experimentally test the PPTs behavior under the described conditions. The band was fixed close to the electrical connections of the PPTs and a sinusoidal displacement was applied at the free end. The motion was imposed by an electrodynamic shaker (mod. S 503, Tira GmbH, Schalkau, Germany) that was fed by a power amplifier (mod. BAA 60, Tira GmbH, Schalkau, Germany). The magnitude and frequency of the imposed displacement were driven by a function generator (mod. 33220 A, Agilent Technologies, Santa Clara, CA, USA) and revealed by a linear displacement sensor (mod. ILD 2200-50, MicroEpsilon, Ortenburg, Germany) pointing along the shaker axis (Figure 11). The exerted force by the shaker was measured by a load cell (mod. 208C03, PCB Piezotronics, Depew, NY, USA) that was mounted in series with the shaker stinger and acquired by a module NI 9234IEPE included in a NI CompactDAQ 9172 (National Instruments, Depew, NY, USA). A 100 kΩ resistor (R) was connected in parallel with each PPT. The signal from each PPT was monitored by a digital oscilloscope (Tektronix, Beaverton, OR, USA, mod. TDS 5054, via a 10 MΩ probe) and acquired by a module NI 9239 through an appropriate front panel via SignalExpress 2015 (National Instruments, Depew, NY, USA). To favor the relative rotation of the free end, a thin layer of beeswax was interposed between the support of the shaker and the band. In accordance with the result of the numerical simulation that is reported in Figure 9c, a set of tests was performed with stationary sinusoidal motion at different frequency (ranging from 1 to 3 Hz in 0.2 Hz steps) and magnitude (ranging from 1.9 to 3.7 mm peak-to-peak in 0.18 mm steps). Each acquisition was performed at a sampling rate of 2 kHz for a duration of 5 s.

## 4. Experimental Results

### 4.1. “Fixed-Point” Conditions

**Figure 12:**
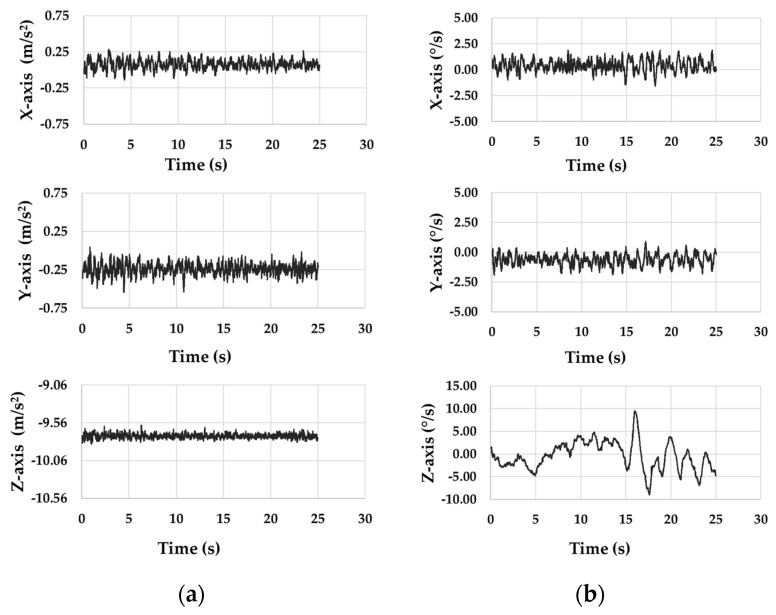
Signals from (**a**) accelerometer and (**b**) gyroscope of iNEMO inertial module.

The spar buoy scaled model was inserted inside an artificial channel, in which the conditions (frequency 2.6 ± 0.05 Hz and amplitude pp 20 ± 1 mm of the wave), as described in Section 2.2, were reproduced. Figure 12a and Table 4 show the acceleration trend and representative values along the three axes from the accelerometer of iNEMO inertial module, respectively. Similarly, Figure 12b and Table 5 highlight the same parameters from the gyroscope of iNEMO inertial module.

As expected, the Z-axis output from the accelerometer of iNEMO inertial module remained constant for the whole test and equal to nearly −9.73 m/s^2^ (Figure 10 and Table 4). It means that the scaled buoy remained integral to the vertical wave motion. The three-axis gyroscope also did not detect appreciable variations in angular oscillations, returning mean values of about 0.40°/s around X-axis and −0.63°/s around Y-axis (Table 5).

### 4.2. PPT as Energy Harvester

**Figure 13:**
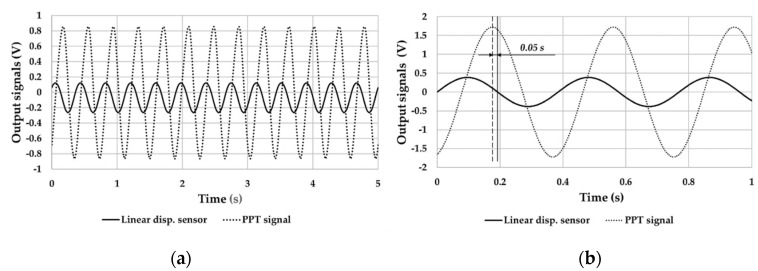
(**a**) Harvester and linear displacement sensor signals over time and (**b**) sinusoidal laws of outputs from PPT and displacement sensor.

Figure 13a exhibits the trend of the data from the linear displacement sensor and the PPT that was placed on the upper side of the band at a frequency of 2.6 Hz (close to the frequency found in sea conditions) and a peak-to-peak magnitude of 1.9 mm.

The sinusoidal laws of outputs from PPT and displacement sensor are shown in Figure 13b. These were calculated while considering the mean values of magnitude and frequency of each signal and the phase between the same signals in the total duration of the test.

PPTs outputs revealed trends with same period of the linear displacement. Furthermore, in the PPTs outputs. no fluctuation in the mean value and in peak-to-peak magnitude was found. The PPT output, related to the band strain, was in quadrature with respect to the imposed linear displacement, as highlighted in Figure 13b. The main reason for this behavior depends on the typical electrical character of the PPT. Therefore, a direct proportionality of the displacement with the strain speed of the band was deduced, in accordance with the scientific literature [27]. In addition to the phase quadrature, a phase shift of 0.05 s was found between the two signals, which was probably due to the viscous contribution provided by the beeswax interposed between the harvester and shaker support.

### 4.3. Reaction Forces Measurement

**Figure 14:**
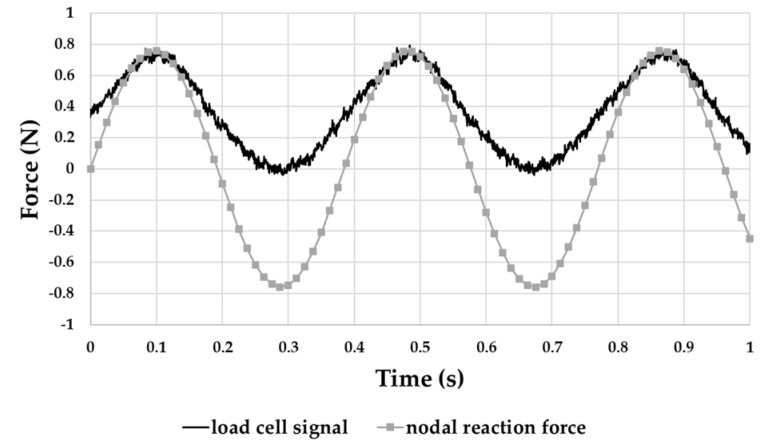
Comparison between load cell output and FEA computed reaction force.

Under the same sinusoidal load condition (frequency of 2.6 Hz and peak-to-peak magnitude of 1.9 mm), as described in Section 4.2, the load cell in series with the shaker stinger provided the trend of Figure 14. Additionally, the values from the FEA (Figure 9d) are indicated.

The band was unable to exert any tensile stress on the load cell due to the way the experimental setup was designed. For this reason, the load cell signal only showed positive sinusoidal fluctuations (Figure 14). Nevertheless, Figure 14 shows a good matching of magnitude and frequency of signals between the experimental and numerical data.

## 5. Data Analysis and Discussion

**Figure 15:**
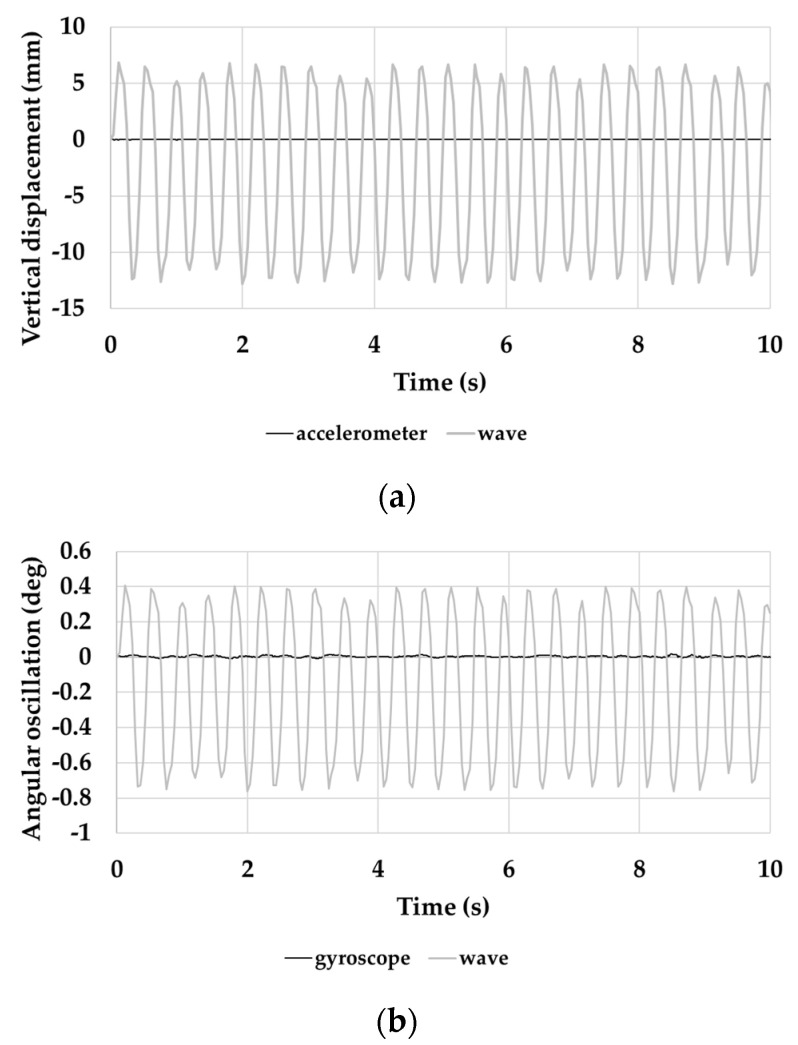
(**a**) comparison between the vertical displacement measured by accelerometer along the Z-axis and the wave magnitude; and, (**b**) comparison between the angular oscillation measured by gyroscope around X-axis and the oscillation occurred by the wave.

The condition of “fixed point” of the buoy with respect to the forced excitation in both vertical and angular oscillation was demonstrated. In effect, Figure 15a shows the comparison between the curve of the wave motion vertical magnitude and that of the vertical displacement (along Z-axis) that was obtained from accelerometric sensor output. The displacement along the Z-axis was obtained through a double integration of the measured accelerometric signal. It has to note that, under a peak-to-peak magnitude excitation of about 15 mm, the buoy undergoes negligible vertical displacements. Similarly, Figure 15b shows the comparison between the angular oscillation of the wave motion (*γ*) and that returned by the gyroscope (around X-axis). The angular oscillation was calculated as:

*γ* = *w_d_* · arctan [(*T*/2)*/L*] (4)

where *T* is the wave period, *L* indicates the barycentric length between the ballast and the buoy, and wd is the wave displacement magnitude.

Based on the signals that were acquired by the PPTs, it was possible to determine an energy forecast of the harvesting operation. The disposable electrical power (*P*) was calculated based on the peak-to-peak voltage magnitudes (*V_pk-pk_*) of the PPT according to Equation (5):

*P* = *V_pk-pk_*^2^*/R* (5)

where *R* was the resistor connected in parallel with the PPT.

Consequently, it was possible to evaluate the storable energy (*E*) through Equation (6):

*E* = *P/f* (6)

where *f* indicates the working frequency.

**Figure 16:**
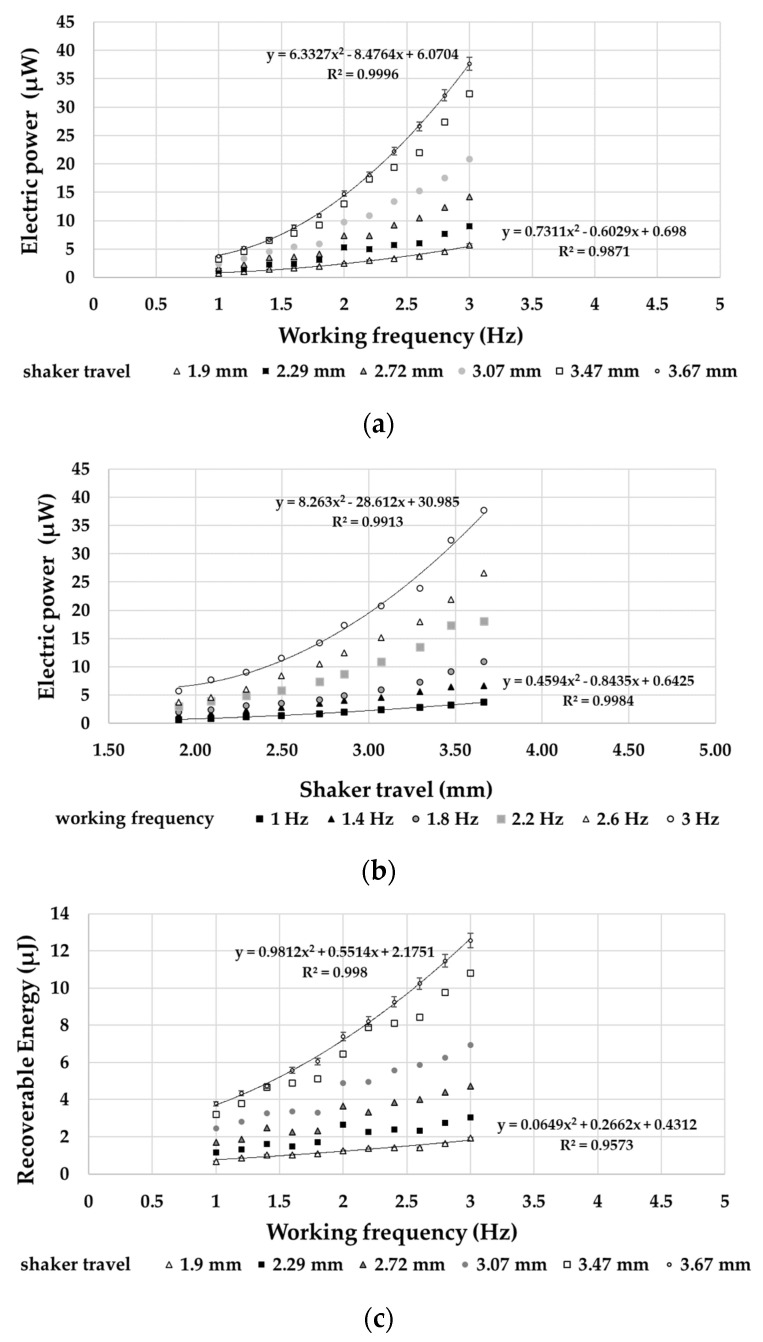
Output power disposable from a single PPT as a function of (**a**) frequency and (**b**) shaker travel, (**c**) energy recoverable from a single PPT.

Electric power values, as shown in Figure 16a and b, denote a monotonous dependence with respect both the magnitudes and frequencies of the imposed motion, with a distinctly parabolic trend line. The energy values, as shown in Figure 16c, exhibit an energy harvesting trend in agreement with the scientific literature [28].

**Figure 17:**
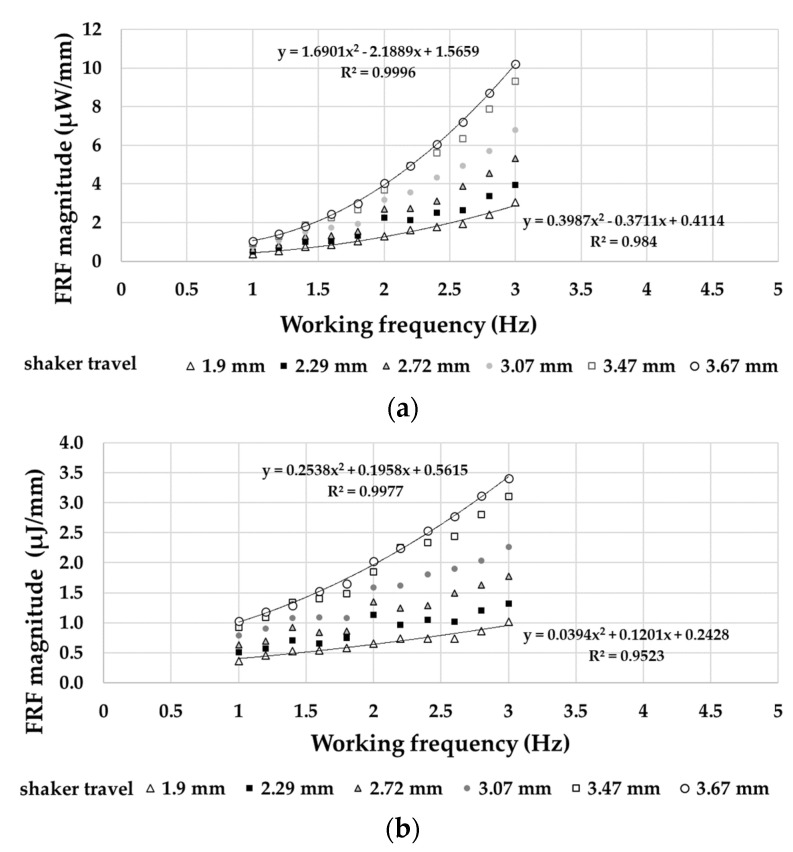
(**a**) Power/linear displacement ratio frequency response functions (FRF) and (**b**) Energy/linear displacement ratio FRF.

An experimental investigation on the frequency response functions (FRF) of the harvester was carried out. In this context, power and energy were chosen, respectively, as the output variable, while the linear displacement that is supplied by the shaker as the input variable. Figure 17a and b display the modules of power/magnitude ratio and energy/magnitude ratio, respectively, as the working frequency changes.

The accuracy of the performed measurements (Figure 16 and Figure 17) corresponds to the deviation of the values of the single experimental points from the relative best-fit curves. This deviation falls within a standard deviation of 3%.

## 6. Conclusions

The paper introduces an innovative design for a self-powered monitoring buoy, which was characterized by the “fixed-point” concept, with the advantage of using wave motion as a source of electricity available for low-powered on-board sensors.

The employed energy harvesting system is performed by means of piezoelectric patch transducers, working in low frequency. In fact, this type of buoy operates between 2 and 3 Hz, which correspond to the maximum frequencies found in the sea, i.e., ripples waves. Such a design allows obtaining a continuous energy source, for example, unlike photovoltaics, which provides a greater power supply, but exclusively dependent on the presence of solar radiation. Therefore, although the amount of energy that is produced by the piezoelectric patches is small, it is still sufficient to feed the on-board sensors through a suitable boost converter and a rechargeable accumulator. It should be considered that, in the face of the intermittent operation of the on-board sensors and the remote data transmission system, the recharge of the accumulator should instead take place continuously (24 h per day).

Future developments will focus on the implementation of a full-size buoy, which will be six times larger than the presented scaled model. For such device, six piezoelectric patches will be mounted on three deformable band, i.e., two patches of each band. Consequently, it will increase the harvested energy by six times. Moreover, although such a buoy will work in near-shore area, the full-size one will be able to withstand waves in an amplitude greater than ripples wave. Further tests will be carried out in order to investigate the behavior of the full-size buoy and of the energy harvesting system at sea. Finally, it is worth noting that the powers and, consequently, the energies harvested, could be increased while using specific interface circuits [29], impedance matching devices and strategies [30,31], and power tracking strategies [32].

## Figures and Tables

**Table 1:** Summary on the buoy unit sizing.

*d*	*S*	*B*	*H*_*e*_	*M*	*L*	*λ*_*vert*_	*λ*_*ang*_
(mm)	(mm)	(N)	(mm)	(kg)	(mm)		
600	500	1052.5	100	57.5	2000	10.62	5.67

**Table 2:** Summary on the buoy model sizing.

*d*	*S*	*B*	*H*_*e*_	*M*	*L*	*λ*_*vert*_	*λ*_*ang*_
(mm)	(mm)	(N)	(mm)	(kg)	(mm)		
100	86	3.98	14	0.45	370	12.73	14.69

**Table 3:** FEA parameters.

FEA Specifications	Measure Unit	Carbon Fiber (band)	Kapton (PPT)	Epoxy Resin (ribs)
**Elastic modulus**	(GPa)	70	2.07	2.415
**Poisson ratio**		0.3	0.39	0.35
**Effective Length**	(mm)	350		
**Width**	(mm)	35		
**Thickness**	(mm)	1		
**Solid Mesh Nodes**		18,746		
**Solid Mesh Elements**		8556		
**Maximum Element size**	(mm)	4.546		
**Tolerance**	(mm)	0.2273		
**Percentage of elements with Aspect Ratio < 3**	(%)	0.409		
**Percentage of elements with Aspect Ratio > 10**	(%)	23.9		
**Maximum Aspect Ratio**		34.565		
**Maximum Jacobian**		10.18		
**Contacts between parts**		bonded		
**Boundary Conditions:**				
**Maximum Remote Displacement**	(mm)	10 (at 350 mm from fixed joint)		
**Simulation Time Duration**	(s)	1		
**Fixed Time Step**	(s)	0.04		

**Table 4:** Data from triaxial accelerometer in iNEMO inertial module.

	Max	Min	Mean Value	St. Deviation
	(m/s^2^)	(m/s^2^)	(m/s^2^)	(m/s^2^)
X-axis	0.28449	−0.13734	0.07633	0.065729
Y-axis	0.04905	−0.53955	−0.2377	0.075556
Z-axis	−9.59418	−9.83943	−9.73372	0.029311

**Table 5:** Data from triaxial gyroscope in iNEMO inertial module.

	Max	Min	Mean Value	St. Deviation
	(°/s)	(°/s)	(°/s)	(°/s)
X-axis	1.9	−1.6	0.401346	0.569683
Y-axis	0.9	−1.9	−0.62611	0.472373
Z-axis	9.5	−9.0	−0.3975	3.103408

## Data Availability

Not applicable.

## Nomenclature

**Symbol**	**Units**	**Description**
*x*	(mm)	forcing excitation function
*X*_0_	(mm)	forcing excitation magnitude
*y*	(mm)	response excitation function
*Y*_0_	(mm)	response excitation magnitude
*g*	(deg)	angular oscillation
*m*	(kg)	mass
*b*	(mm^2^/s)	cinematic viscosity
*β_cr_*	(mm^2^/s)	critical viscosity
*k*	(N/mm)	stiffness
*ω*	(rad)	pulsation
*ω*_0_	(rad)	natural pulsation
*φ*	(rad)	phase of the transfer function
*λ*		pulsation ratio
*τ*		viscosity ratio
*g*	(m/s^2^)	gravity acceleration
*t*	(s)	time
*T*	(s)	wave period
*f*	(Hz)	wave frequency
*M*	(kg)	spar buoy or scale model mass
*d*	(mm)	spar buoy or scale model diameter
*S*	(mm)	buoy sinking
*He*	(mm)	emerged height of the spherical cap
*L*	(mm)	barycentric length between spherical cap and ballast
*B*	(N)	Buoyancy
*λ_vert_*		vertical oscillations pulsations ratio
*λ_ang_*		angular oscillations pulsations ratio
*w_d_*	(mm)	wave magnitude
*I*		experimental setup signals generator
*O*		experimental setup signals acquisition
*o*_1_		linear displacement signal
*o*_2_		load cell signal
*o*_3_		PPT voltage signal
*R*	(kΩ)	load resistance
*Z*	(MΩ)	load impedance
*V_pk_* *_-pk_*	(V)	PPT peak-to-peak voltage
*P*	(mW)	harvested electric power
*E*	(mJ)	harvested electric energy
